# Customer mobility signatures and financial indicators as predictors in product recommendation

**DOI:** 10.1371/journal.pone.0201197

**Published:** 2018-07-27

**Authors:** Cagan Urkup, Burcin Bozkaya, F. Sibel Salman

**Affiliations:** 1 Department of Industrial Engineering, Koç University, Istanbul, Turkey; 2 School of Management, Sabancı University, Istanbul, Turkey; University of Toronto, Rotman School, CANADA

## Abstract

The rapid growth of mobile payment and geo-aware systems as well as the resulting emergence of Big Data present opportunities to explore individual consuming patterns across space and time. Here we analyze a one-year transaction dataset of a leading commercial bank to understand to what extent customer mobility behavior and financial indicators can predict the use of a target product, namely the Individual Consumer Loan product. After data preprocessing, we generate 13 datasets covering different time intervals and feature groups, and test combinations of 3 feature selection methods and 10 classification algorithms to determine, for each dataset, the best feature selection method and the most influential features, and the best classification algorithm. We observe the importance of spatio-temporal mobility features and financial features, in addition to demography, in predicting the use of this exemplary product with high accuracy (AUC = 0.942). Finally, we analyze the classification results and report on most interesting customer characteristics and product usage implications. Our findings can be used to potentially increase the success rates of product recommendation systems.

## Introduction

Digital technologies have led to the rapid growth and increasing availability of Big Data. While millions of data records accumulate on a daily basis, most businesses face the same struggle: how to extract beneficial information from these data. Businesses are lately investing in systems that can analyze their databases and uncover hidden patterns through various technical approaches. The better understanding of their customers’ choices and preferences improve their business decisions, which leads to a better competitive advantage and increased profits. However, the sheer size of the transactional databases reduces the efficiency and quality of recommendations. On the other side, the customers also struggle while choosing from an ever increasing variety of products under excessive information. Rodrigues and Ferreira [1] point out that the increase in product diversity directly challenges the businesses to offer the right products to the right customers. This is where recommendation systems come into picture and cope with this challenge. Recommendation systems provide suggestions to the customers according to customers’ preferences that are obtained through data analysis [2]. A recommendation system exploits the hidden patterns in Big Data and simplifies the recommendation process for businesses. As a result, customers are not exposed to products that they are not expected to buy, which also simplifies the process from the customers’ perspective.

The literature on product recommendation focuses on various types of Big Data including financial status and purchase history of customers in order to recommend the best products [3], [4]. Moreover, there exist other studies on location predictability of people that use mobility patterns of customers via digital traces. For instance, Noulas et al. [5] use Foursquare check-in data to predict the next locations of the users. In fact, any dataset including location and time stamp information may lead to understanding individual mobility patterns of users, which could lead to taking various business actions such as designing location-based marketing campaigns, new product recommendation, and preventing fraudulent activities on customer accounts.

Digitalization of customer spendings and developments in mobile banking have turned banks into one of the most prominent Big Data producers. The datasets that are potentially available from banks possess various types of data including demographic, financial and mobility information about customers, but without an effective analysis of such data, the data does not mean anything alone [6]. The existence of mobility dimension creates tremendous new opportunities in the field of data analytics, which is ever developing due to the availability of large-scale datasets reflecting human interactions, communications and mobility [7].

In this article, we study a one-year (July 2014-June 2015) customer transaction dataset of a leading bank in Turkey to investigate the predictive power of customer mobility patterns and financial indicators on the use of a target product. After data cleansing and formatting, we seek a relation between product usage and customer mobility signatures, employing the features we have designed. The target banking product we have selected to apply our approach is the Individual Consumer Loan (ICL) product, which is one of the most profitable and active products of the bank. After we apply the best feature selection and classification algorithms to our datasets, we discover that the features we design and derive from mobility signatures of individuals as well as features on financial activity demonstrate statistical significance in predicting ICL product usage. Hence, the main contribution of our study lies in the discovery that the performance of product recommendation systems can be improved significantly using the financial features as well as mobility features we propose in this study in addition to demographic features. Furthermore, we note that while some of the features we use have appeared in other recent studies in different contexts, our study, to the best of our knowledge, is the first one that combines these features in the context of product recommendation.

The rest of the article is organized as follows. We provide the relevant background and literature in the next section. We then describe our dataset and the preprocessing steps we have implemented. After presenting the performance evaluation criteria, feature selection/extraction methods and classification algorithms, we report the results of our computational experiments and point out our deductions. Finally, we summarize our main contributions and the inferences we obtain from our study.

## Literature review

In this section, we provide a review of the literature on product recommendation and analysis of customer mobility behavior separately. Although each one of these topics is extensively studied in the literature, there does not exist, to the best of our knowledge, research on product recommendation as related to both an individual’s mobility signatures and financial activity.

In product recommendation systems, several studies have attempted to connect an individual’s product usage to her demographic data and product similarity measures [8], [9]. However, other than past product usage, we find no publicly reported work to have considered fine-grain “microdata”, such as mobility data, that studies the individual’s behavior in different dimensions. Choi et al. [8] create a system where customers input their preference via an interface and customers’ similarity measures are calculated with these inputs. The similarity measure of the products are also calculated and the system recommends a list of products to the customer. The authors use a multi-attribute decision making approach to find the utility value of each product.

Park and Chang [9] propose a new weighted customer profiling model, using product features as well as individual and group interests. Then, they generate product groups according to their corresponding similarity measures. They calculate the similarity between a customer and each product group and recommend the most highly ranked *k* products according to the similarity measures.

Use of collaborative filtering in product recommendation is fairly common. Riyaz and Varghese [2] apply their collaborative filtering approach to recommend products on Amazon’s data. The authors start with extracting user preferences from the data. Then, they find similar items based on the user preferences, followed by the final recommendation of products to customers. They use Pearson’s correlation coefficient to calculate the similarity between the products. Liu and Shih [4] use Customer Lifetime Values (CLV) in banking, an estimation of how much profit the customer will bring to the bank, to recommend products to their customers. They evaluate the CLV values according to a weighted Recency, Frequency and Monetary (RFM) combination. The extracted frequent purchase patterns represent the common purchasing behavior of customers with similar product purchases. Therefore, their approach recommends products to customers according to the frequency of purchase patterns of customers with similar product purchases.

Another study on product recommendation is by Sanchez-Moreno et al. [3], who propose a collaborative filtering method for music recommendation using playing coefficients they generate for artists as well as users. They mainly focus on “gray sheep” customers, who cannot directly be assigned to a customer cluster because they show different preferences. Their proposed algorithm is also designed for the case where there is only limited information available, because they claim that the user item and user preference information is not generally available. Their strategy is to determine a coefficient for every user representing the degree to which they are gray sheep users. The authors prove that their approach significantly outperforms other collaborative filtering approaches in the literature in terms of ROC curve and MSE errors.

Use of spatio-temporal data and many resulting types of features is a relatively new approach in data analytics. We see location and time traces being used in a number of studies in predicting output variables or in classification of labels in a variety of contexts. For instance, in a study that is closely related to ours, Singh et al. [10] extract individual behavior from spatio-temporal attributes available in bank transaction data and link it to financial well-being. They process credit card transaction data to generate features based on spatio-temporal aspects of the customer shopping activity. They perform bootstrap aggregation with the SMOTE approach as their classifier and show that these features have high performance in classifying customers with financial performance (AUC = 0.77-0.83).

Gonzalez et al. [11] study the trajectory of 100,000 anonymous mobile phone users whose positions are tracked for a six-month period. They observe that in contrast with the random trajectories predicted by the prevailing Le’vy flight and random walk models, human trajectories show a high degree of temporal and spatial regularity. Here, each individual is characterized by a time-independent travel distance and a significant probability to return to a few highly frequented locations. The authors also calculate the *radius* *of* *gyration* for each user and conclude that the trajectories become more anisotropic as the radius of gyration increases. Similarly, Isaacman et al. [12] use anonymous and aggregate statistics of approximate locations of cell phones in New York and Los Angeles to demonstrate different mobility patterns in these two cities. They introduce a daily range concept that is the maximum distance a person travels in one day. They calculate the daily ranges for each day of the data, and extract median and maximum daily ranges for each customer. They conclude that people living in LA region tend to travel on a regular basis almost 2 times farther than New Yorkers. On the other hand, the maximum distance that a New Yorker travels is 4-5 times greater than people live in LA.

Sobolevsky et al. [7] study bank card transaction data of BBVA, the second largest bank in Spain, to examine how the mobility patterns vary by nationality. They first partition the customers into two groups, Spaniards and non-Spaniards, using an optimization algorithm. Afterwards, they create a network of regions where links represent connections between a customer’s home region and the region where she transacts, and are getting stronger proportional to the amount spent. They use *gravity* *law* *approximation*, which produces a measure combining the weights and the distances between two nodes in the network. They conclude that the Spaniards are more local travelers, while the non-Spaniards tend to travel longer distances within the country.

Krumme et al. [13] study the predictability of customers’ visitation patterns given their economic transaction data. In doing so, they calculate the *temporally* − *uncorrelated* *entropy*, which is the entropy of the store visitation of customers neglecting specific ordering of visits, and the *sequence* − *dependent* *entropy*, which is the compressibility of the sequence of stores. They observe that both entropy distributions are close to each other. They conclude that the sequence-dependent entropy decreases with low temporal resolution.

Based on our literature review, we find, to the best of our knowledge, no research that links product recommendation to individuals’ mobility signatures and financial indicators, hence the novel contribution of our study. In the subsequent sections, we demonstrate the use of appropriate methodologies to reveal how mobility signatures and features on financial activity relate to effective product offerings to be used in product recommendation systems.

## Data and preprocessing

In this section, we present the details of our dataset and explain the features we extract from the dataset. Subsequently, we summarize the data preprocessing methods we perform on the dataset for a better classifier performance. We then discuss the data separation process we implement to observe the differences between different training and testing datasets. Our dataset can be accessed from https://figshare.com/s/99d41cf2962fa77c335c.

### Data

We analyze a one-year customer transaction dataset of a leading bank in Turkey. To anonymize the dataset, customer name and unique identifier (e.g. the social security number) fields have been pre-excluded from the dataset; only pseudo-unique customer identification numbers are provided by the bank. The dataset includes demographic, financial and behavioral information about the customers. It covers key banking transactions belonging to 100,000 customers that occurred in a one-year period, represented by 14 tables, 217 columns and 52,267,342 rows in total. These 14 tables cover various types of banking activity, such as credit card transactions and payment of credit card statements, ATM and money transfer activity, call center calls, bank product ownership and product campaign offerings, web platform activity and branch visit activity. The tables we use in our study include the following:

- ATM (Automatic Teller Machine) transaction table includes transactions such as withdrawal, deposit and money transfers with location data.
- Crosswise sales table represents the activity and ownership of bank products used by customers.
- Call center table includes all call center call information about the customers, including frequency and the number of the operations performed during the calls.
- Electronic funds transfer table includes all the transfers going out of the customer’s account including transfer amounts.
- Remittance table includes the remittance transactions including amount and currency information.
- There are two campaign tables that include all the campaigns that the bank has offered to the customer, the offering channel and the customer responses.
- Credit card receipt table consists of credit card balance and last payment date.
- Credit card receipt payment table includes total credit card debt and the corresponding currencies.
- Credit card expenditure table contains all the credit card expenditures with corresponding amounts, locations, currencies and spending category.
- Credit card information table is comprised of the number of credit cards that the customer owns including other banks and also credit card limits of the customer, again including both our bank and other banks.
- Web banking table includes all transactions that the customer performs via the web platform, including the type of the transaction, currency, channel, amount and total number of transactions.
- Customer information table includes some demographic and financial information about the customers, such as age, gender, occupation, marital status, income, education, bank age and risk scores.
- Auto payment table includes the amount and the total number of auto payments that the customer uses.
- Branch table consists of the number of visits that the customer made to the branches, including location data and the total number of transactions.

### Features

The features that we use are divided into three main categories: demographic, financial and mobility. We provide a list of the features in S1 Table, together with their explanations. The spatio-temporal dimensions of the mobility features, for each customer, are measured based on three notions, similar to as introduced by Singh et al. [10], which are generated with four variations of “bins” as described below.

**Diversity** is a measure of customer’s shopping behavior that represents variability over space and time, measured through “bins” as buckets of space or time into which shopping transactions are tallied. The basic concept is that the more spread out the transactions are into different bins, the more diverse a customer is. Let the fraction of transactions that fall within bin *j* for user *i* be denoted as *p*_*ij*_. We calculate the parameter *p*_*ij*_ from the data for each user independently. We then calculate the spatial (temporal) diversity of user *i* as the normalized entropy of all transactions tallied in all spatial (temporal) bins. Diversity features measure between 0 and 1 and higher diversity values correspond to values closer to 1. That is, a customer with high spatial (temporal) diversity spreads her transactions almost equally across different locations (time buckets).

**Loyalty** refers to the percentage of transactions that occur over a customer’s *k* most frequented bins. Given the same definition of bins, we use *k* = 3 in our study. Let *f*_*i*_ denote the combined fraction of all transactions of user *i* that occur in top 3 most frequented bins. The values of the loyalty features range between 0 and 1; a large loyalty value indicates a higher percentage of transactions in top *k* bins, hence higher loyalty behavior.

**Regularity** measures how repeating a customer’s diversity and loyalty behavior is over a period of time (month, week, etc.). We use the month as our unit period and divide the overall time frame into two: the observation period where we calculate diversity and regularity features, and the comparison period where we check if the observed behavior (i.e., the calculated values) repeats. For instance, for a 9-month regularity measure, we observe for the first 6 months and compare with the last 3 months of activity. The regularity features also measure between 0 and 1; the closer the values to 1, the more repeating the pattern in the last 3 months. In our analysis, we consider, in addition to the 9-month time frame, 11-month, 6-month and 3-month regularity measures as well. The observation periods for these are 8, 4 and 2, respectively. In all of the cases, the 12th month corresponds to the label to be classified.

We create the bins according to two interpretations for space and two for time:

Temporal-Hourly: The time period is taken as 8-hourly, i.e., each bin spans 8 hours. We use 12am-8am, 8am-4pm and 4pm-12am as the three bins. The reason is that aggregating the transactions into larger time periods results in better prediction performance than using 1-hour bins.
Temporal-Weekly: The time period is taken as weekly. For example, 35*^th^* and 20*^th^* weeks of the year are two bins.
Spatial-Radial: A radial structure is implemented where the bins are concentric circles with 1, 3, 5, 15, 50, 150 and 500 kilometer radii. We use the center of gravity of all transaction coordinates of a customer as the center of the radial structure for that customer. The center of gravity provides strong information about the customer’s most frequented location. Therefore, this spatial radial measure indicates how far the customer travels from her most frequented position.
Spatial-Cluster: A clustering structure is applied and the transactions are assigned to 50 clusters across Turkey. For example, the cluster center in Istanbul and the center in Ankara are major cluster seeds because of the excessive number of transactions around those coordinates.

### Data preprocessing

The very first step in data preprocessing is typically the handling of null data points. Null data distorts feature selection as well as prediction algorithms [14]. Various algorithms are offered in the literature to replace missing values [15]. As Kim et al. [16] report, missing value replacement approaches try to make strong estimations for the missing values, but this process should be performed accurately, because changing the data points directly affects the prediction results. Even then, missing value replacement is a perturbation of the original data. For these reasons, we choose to simply remove the records with missing data elements, which correspond to less than 1% of our data.

The other preprocessing step we implement is to transform all categorical features into numeric ones. We do this by turning each categorical value into a column of binary values. For instance, the gender column is transferred into male and female columns, where male customers have 1 in the male column and 0 in the female column. This process allows classification algorithms to use the data in numeric format, however it increases the number of features to 90.

**Fig 1:**
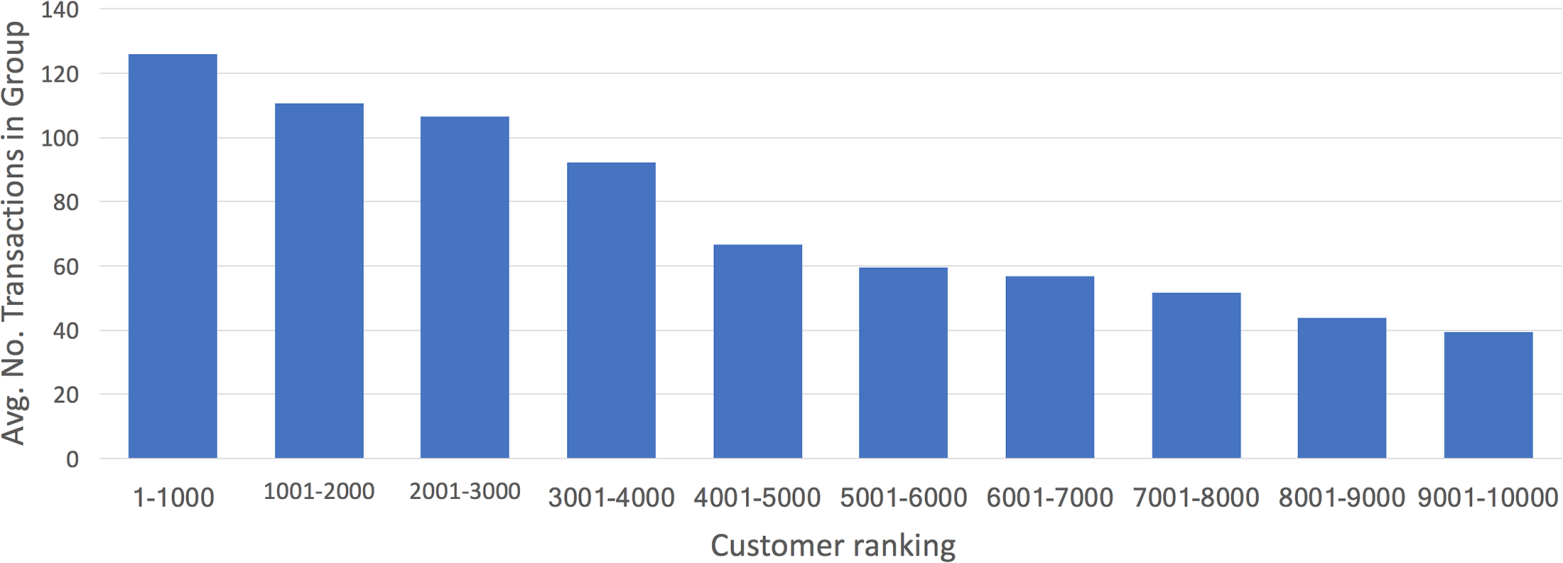
Percentile distribution of top 10,000 customers’ transaction counts.

Finally, we apply a detailed filtering scheme on customers to obtain sets of more desirable customers. The data include all transactions of 100,000 customers, with various age, job, income, etc. distributions. Some customers are very active with frequent transactions, and some are not. We choose to filter these 100,000 customers into a smaller subset, which we think is more useful for the objectives of our study. We first filter out customers whose total number of credit card transactions over 12 months is 10 or less. We also filter out customers whose bank POS (Point of Sale) transaction rate (for transactions completed on the bank’s own POS machines) is less than 60%. We further filter out customers whose monthly income is less than 500 *TL* (Turkish Lira) and greater than 300,000 *TL*. After these steps, we sum the number of credit card transactions and the number of ATM transactions with location stamp for all customers, sort them in descending order of this sum, and select the top 10,000 customers. Since one objective of our study is to reveal how effective customers’ mobility behavior is in predicting product usage, filtering the customers in this manner will aid the study in line with our objectives. Table 1 shows the descriptive statistics of combined credit card and ATM transactions for the top 10,000 customers and Fig 1 depicts the percentile distribution of the same sorted from most frequent to less frequent transactions. Note that even though we first filter out customers with 10 transactions or less, the final set of customers we consider still have a lot more transactions (75.2 overall average, and 125.8 average for the top 10 percentile).

**Table 1:** Descriptive statistics for the sample data.

**Mean**	75.2
**Std. Dev.**	46.8
**Min**	34.0
**25% percentile**	44.7
**50% percentile**	65.1
**75% percentile**	100.9

### Dataset separation

After the preprocessing steps, we gather all required features and filtered customer records in one table by processing 14 different data tables. To understand the impact of data recency, we create 4 versions of our data table by choosing monthly intervals for months 9-11 (most recent 3 months), months 6-11 (most recent 6 months), months 3-11 (most recent 9 months) and months 1-11 (11 month data). We use the target product usage in month 12 as our classification label. To understand the impact of having different feature types, we also separate the features into four groups: demographic features only, demographic+mobility features, demographic+financial features, and demographic+mobility+financial features. This gives us a total of 13 combinations, resulting in the datasets listed in Table 2.

**Table 2:** Dataset explanations.

Dataset	Transactions Spanning Months	Feature Types
**D**	Does not Depend on Month	Demographic
**11DM**	1-11	Demographic & Mobility
**11DF**	1-11	Demographic & Financial
**11DFM**	1-11	Demographic & Financial & Mobility
**9DM**	3-11	Demographic & Mobility
**9DF**	3-11	Demographic & Financial
**9DFM**	3-11	Demographic & Financial & Mobility
**6DM**	6-11	Demographic & Mobility
**6DF**	6-11	Demographic & Financial
**6DFM**	6-11	Demographic & Financial & Mobility
**3DM**	9-11	Demographic & Mobility
**3DF**	9-11	Demographic & Financial
**3DFM**	9-11	Demographic & Financial & Mobility

The features are calculated independently for the datasets spanning different monthly intervals. For example, the number of active products column for 3DF considers the banking activity in months 9 through 11; the number of active products column for 6DFM considers activity in months 6 through 11, and so on. After producing 13 different tables with the corresponding features, we apply normalization to reduce data redundancy and to improve data integrity [17]. We implement max-min normalization [14]. Eventually, our data tables are ready for Feature Selection/Extraction (FSE) and classification methods.

## FSE methods and classifiers

We now present our approach in selecting the best FSE methods and the best classification algorithms for our analysis. We first clarify how we evaluate the performance of the FSE methods, and how we implement the FSE and classification methods with each of our 13 datasets independently. Afterwards, we briefly explain all of the FSE methods and the binary classifiers we utilize. In our study, the classification algorithms indicate whether a customer will use the target product in month 12 or not.

### Performance evaluation

We follow a simple logic used by Chandrashekar and Sahin [18] in evaluating the performances of the FSE methods: the better the classifier performs, the better the FSE is. This argument is reasonable since the information transferred to the classification algorithm is determined by the FSE method. Therefore, we use the performance of the classification algorithms to interpret the performance of different FSE methods. There are five common performance evaluation metrics in data analytics: accuracy, area under the ROC (receiver operating characteristic) curve (AUC), precision, recall and F1, which we use in all performance evaluation steps for various FSE methods. Since there are five metrics, we take the average of five scores and and use it to determine the best performing methods.

### Feature selection and extraction (FSE) methods

The concept of feature selection is essentially about selecting a most relevant and effective subset of the input set of variables in the dataset to be used as predictors. Achieving success in the classification process requires choosing the best classification algorithms using best FSE methods [18] and in our case, this is done for each dataset independently. The reason for independence is that the datasets contain different subsets of the features with different values due to the time intervals. For instance, applying a FSE algorithm on the dataset with the 3 month interval including mobility features may result in a different performance from applying the same algorithm on the dataset with the 9 month interval without mobility features. We use 10 classification algorithms and compare their average performances for each FSE method.

We consider three FSE algorithms in our analysis: f_regression, Recursive Feature Elimination (RFE) and Principal Component Analysis (PCA). Each algorithm works in a different way and with each, we try to find the best features that explain as much of the variability in the datasets as possible [19]. For each of the 13 datasets, we take 5 runs (with small changes on classifiers’ parameters) using a 10-fold cross validation technique as mentioned in [20]. We use *k* = 10 because this does not increase the running times excessively and 10 random training/testing samples are sufficient to eliminate data dependency. The split ratio we use in the study for training and testing samples are 7000 and 3000 customers, respectively.

Finally, we analyze the performance of each FSE method on each dataset and we compare these values to obtain the best method for each dataset. For instance, for the 9DFM dataset, we compare the average classifier performance of all FSE methods and select the best FSE method for this particular dataset as RFE. Afterwards, we determine the number of features and the corresponding FSE methods that have the best performance. For instance, after we find that RFE performs best for the 9DFM dataset, by using the same evaluation metrics, we determine that the best performing number of features for this scenario is 40. The final step is to determine the best classification algorithms using the results of the FSE process.

### Classification algorithms

We implement 10 classification algorithms listed below with various characteristics and parameters to find the most appropriate ones for our study under each dataset. All 10 classifiers are implemented using libraries in http://scikit-learn.org.

- Logistic regression classifier (Log)
- Decision tree classifier (DT)
- Random forest classifier (Rand)
- Extra trees classifier (ET)
- OneVsOne multi-class classifier (OVO)
- OneVsRest multi-class and multi-label classifier (OVR)
- Error correcting output code classifier (ECOC)
- Stochastic gradient descent classifier (SGD)
- Gaussian Naive Bayes classifier (GNB)
- Multi-layer perceptron neural network classifier (NN)

To determine the best classifiers for each dataset, we first fix the (dataset, FSE method, number of features) combination that we obtain, and we apply all the classifiers listed above to each dataset. Afterwards, we analyze the results and determine the best dataset-classifier assignments. Then, we fix all the information we have and replicate the analysis for 10 iterations with different classifier parameters. Eventually, we obtain the best performing (dataset, FSE method, top three features, classifier, classifier parameter) setting.

### Final model testing

As a final step, after obtaining the best FSE-classifier assignments and related parameter settings, we conduct a final test using our models on a completely different subset of 3,000 customers. This step is taken in order to show that our models have not been overfitting, and the same level of model performance can be achieved consistently. The 3,000-customer set we use involves those customers who come after the initial 10,000 in the ordered list obtained during data preprocessing.

## Computational results

We now present the results for predicting usage of the Individual Consumer Loan (ICL) product. We test all combinations of the FSE methods and classifiers listed in the above section with each dataset, and obtain the results that correspond to the best FSE-classifier choices.

### Analysis of the results

**Fig 2:**
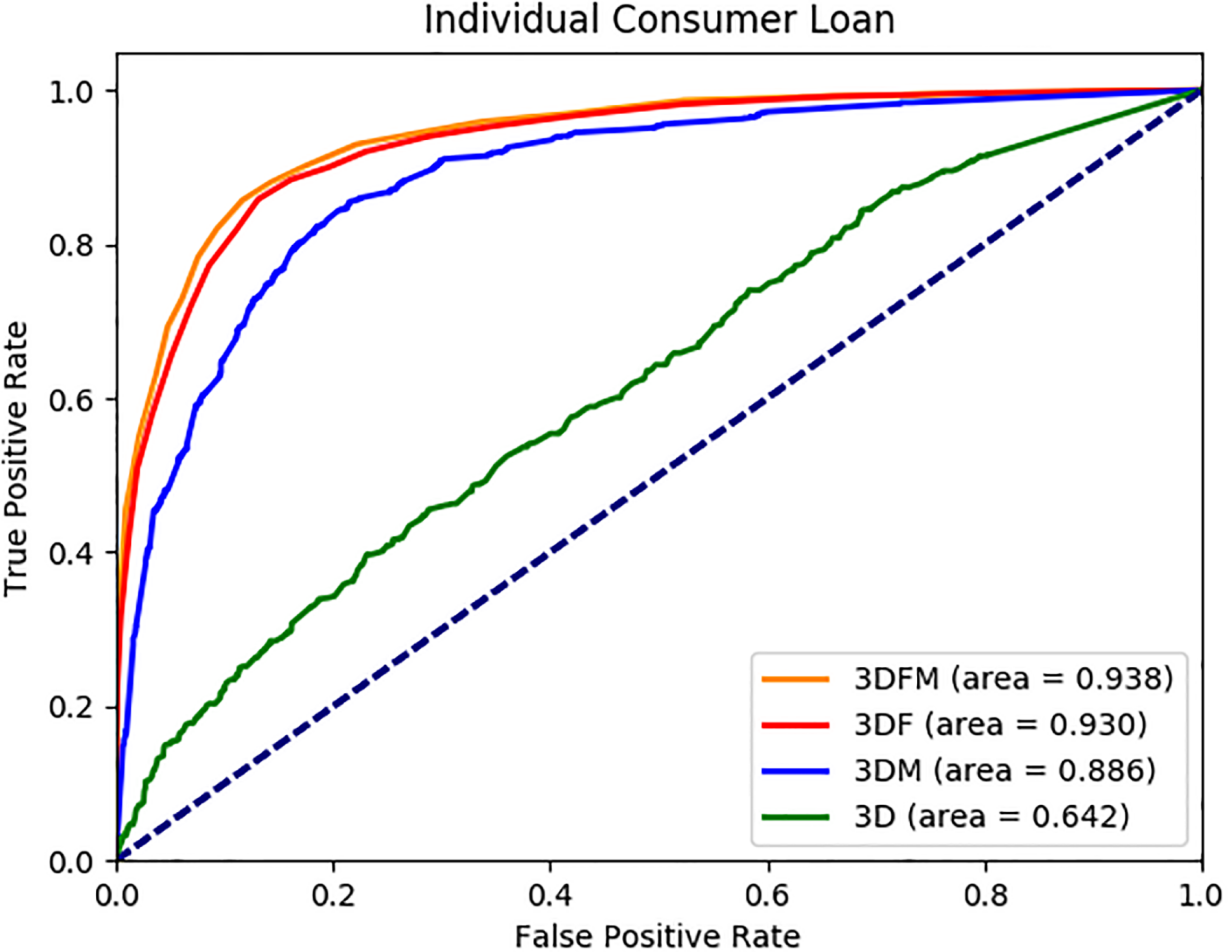
ICL 3 month AUC results.

**Fig 3:**
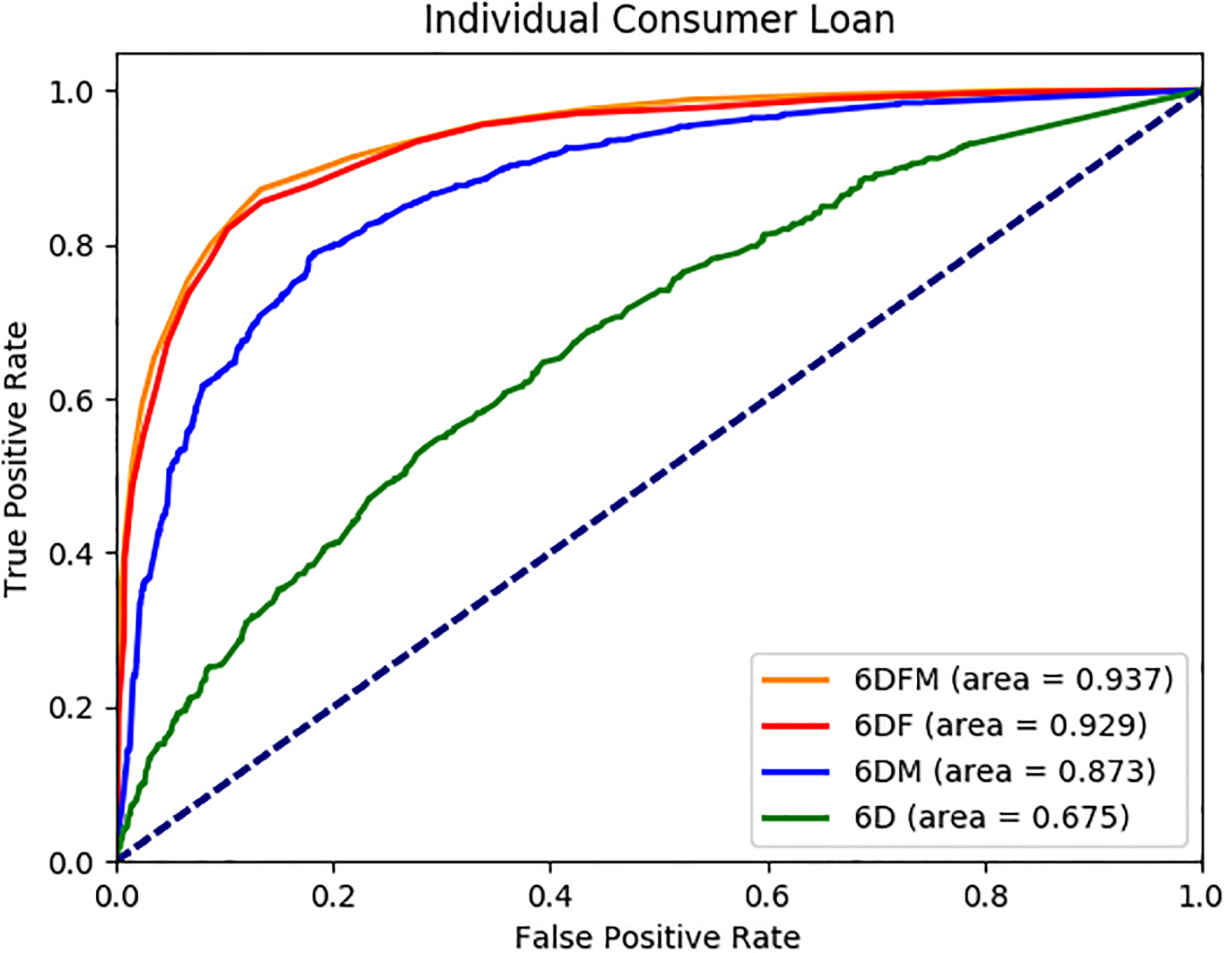
ICL 6 month AUC results.

**Fig 4:**
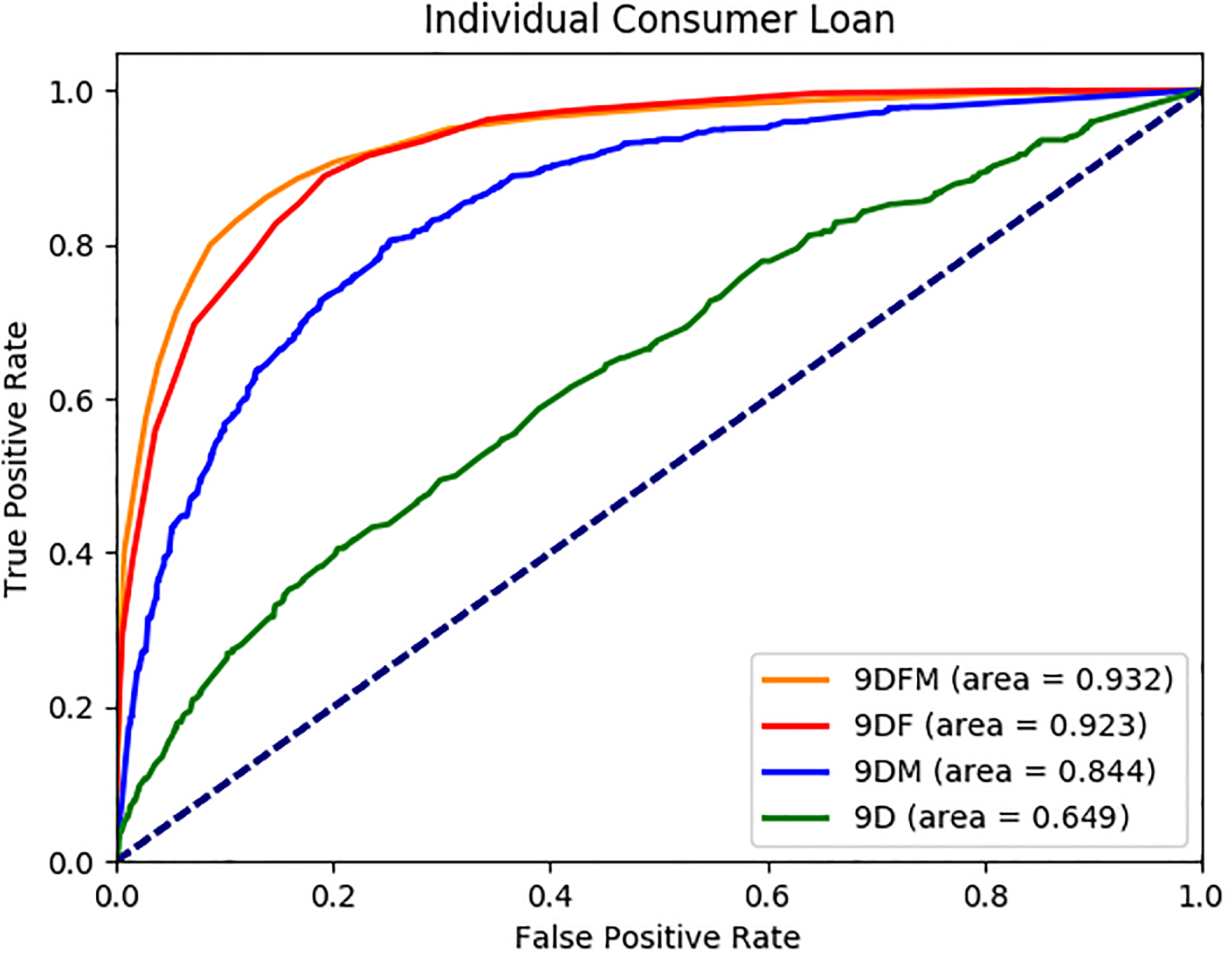
ICL 9 month AUC results.

**Fig 5:**
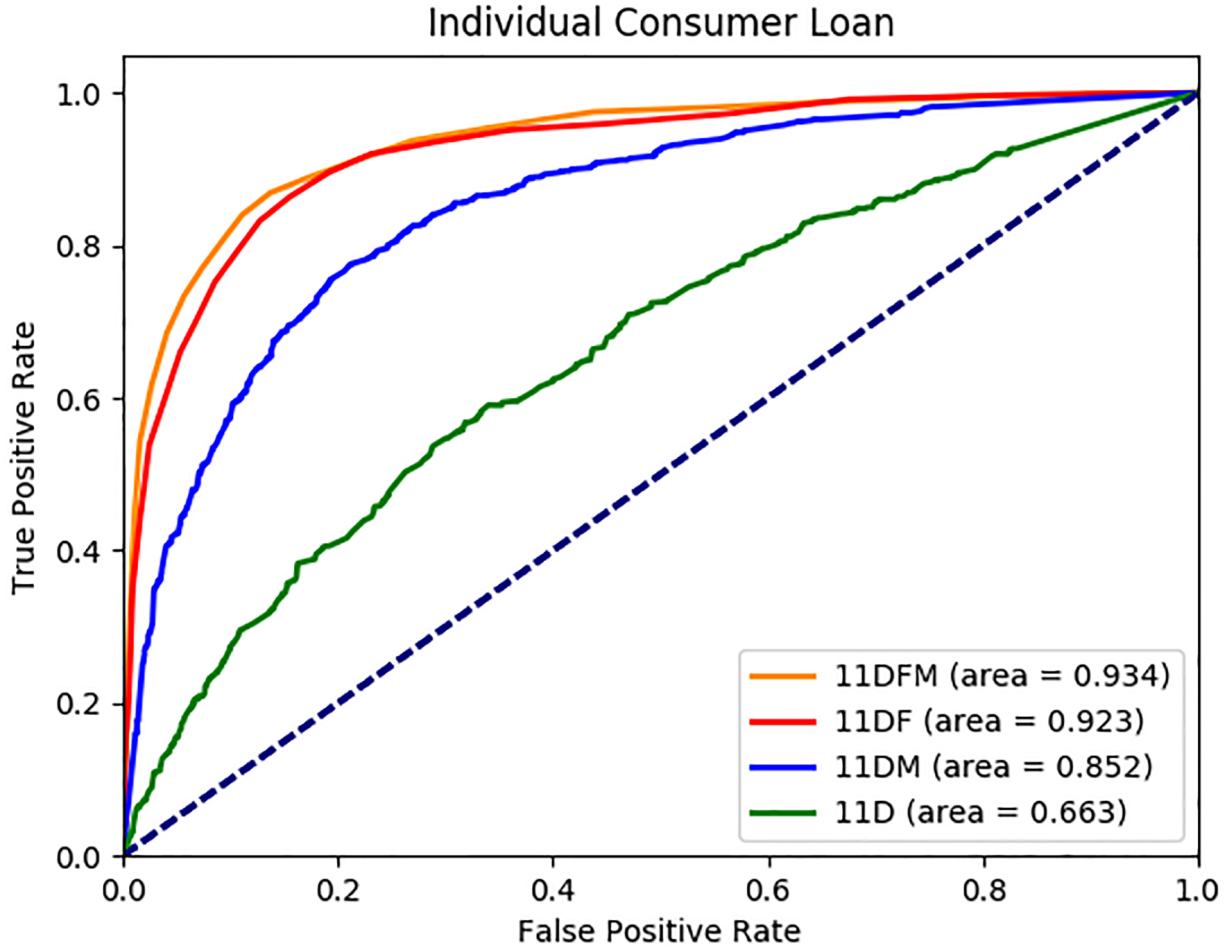
ICL 11 month AUC results.

We report the average AUC, accuracy, precision, recall and F1 values in Table 3 for each dataset. Sample ROC graphs are shown in Figs 2 to 5. The results reported in Table 3 suggest that the FSE and classifier approaches selected under each dataset perform quite well in terms of AUC, accuracy, precision, recall and F1 measures, with average values across 13 datasets of 0.89, 82%, 0.80, 0.76 and 0.78, respectively. All the measures are important to evaluate the performance of the methods, because we have balanced data where 44% of the customers use the ICL product in month 12. Furthermore, the ROC graphs of the 13 datasets as shown in Figs 2 to 5 suggest that the performance ranking of the feature types we use is *DFM* ≥ *DF* ≥ *DM* ≥ *D*, where ≥ denotes equal or better performance.

**Table 3:** Performance measures for different datasets.

Performance Measures					
Dataset	AUC	Accuracy	Precision	Recall	F1
**D**	0.669	62.431	0.537	0.646	0.587
**11DM**	0.835	80.233	0.799	0.636	0.696
**11DF**	0.918	82.856	0.874	0.767	0.817
**11DFM**	0.924	86.922	0.858	0.828	0.843
**9DM**	0.847	79.067	0.739	0.713	0.715
**9DF**	0.924	86.056	0.850	0.787	0.817
**9DFM**	0.931	87.211	0.846	0.802	0.823
**6DM**	0.869	79.733	0.761	0.683	0.709
**6DF**	0.931	85.456	0.843	0.821	0.832
**6DFM**	0.940	85.651	0.838	0.854	0.846
**3DM**	0.878	79.500	0.767	0.715	0.730
**3DF**	0.932	86.056	0.855	0.812	0.833
**3DFM**	0.942	85.556	0.868	0.830	0.849

To show that the performance differences between feature sets are indeed statistically significant, we extend our cross-validation for the 9-month datasets and conduct 30-fold cross-validation followed by paired t-tests. Table 4 shows the *p*-values for all pairwise comparisons between 4 feature sets (D, DM, DF and DFM) for all 5 performance measures (AUC, accuracy, precision, recall and F1). The results indicate that the ordering *DFM* ≥ *DF* ≥ *DM* ≥ *D* is indeed statistically significant in all performance measures. We have chosen the 9-month dataset over the others because the marginal contribution of the mobility features over DF is almost the lowest and 9 months is a reasonably medium length of period, compared to others, for making predictions.

**Table 4:** Results of t-tests (p-values) for comparison of mean performance measures.

**Accuracy**	**9DM**	**9DF**	**9DFM**
**D**	2.76E-40	1.33E-47	1.33E-50
**9DM**		2.21E-40	2.96E-39
**9DF**			7.85E-10
**AUC**	**9DM**	**9DF**	**9DFM**
**D**	8.20E-21	6.14E-25	1.78E-27
**9DM**		5.21E-05	5.78E-11
**9DF**			2.23E-06
**Precision**	**9DM**	**9DF**	**9DFM**
**D**	2.25E-49	4.87E-50	4.18E-53
**9DM**		3.68E-31	2.32E-34
**9DF**			1.30E-10
**Recall**	**9DM**	**9DF**	**9DFM**
**D**	7.07E-13	1.21E-39	4.99E-45
**9DM**		4.28E-46	9.78E-49
**9DF**			3.98E-29
**F1**	**9DM**	**9DF**	**9DFM**
**D**	4.21E-44	6.36E-51	4.13E-57
**9DM**		1.27E-43	1.68E-48
**9DF**			1.94E-25

The results in Table 3 further suggest that as the monthly buckets decrease (i.e., from 11 months to 3 months), the overall performance of the classifiers increases and the difference between feature groups DFM and DF increases. We find that, in terms of performance comparison, 3*DFM* ≥ 6*DFM* ≥ 9*DFM* ≥ 11*DFM*, 3*DF* ≥ 6*DF* ≥ 9*DF* ≥ 11*DF*, and 3*DM* ≥ 6*DM* ≥ 9*DM* ≥ 11*DM*. Moreover, we find that (3*DFM* − 6*FDM*) ≥ (6*DFM* − 9*FDM*) ≥ (9*DFM* − 11*FDM*), where the − operator denotes the perceived performance difference between two feature sets. These findings suggest that as we consider more recent mobility behavior with shorter time frame, we obtain better classifier performance. Clearly, 11 months of activity may be misleading if a customer has shifted patterns along the way.

We also calculate the relative importance of each feature as listed in Table 5. The importance of a feature is the increase in the prediction error when that specific feature is excluded from the model and all the other features are kept. Liaw and Wiener [21] use this approach and try to understand the importance of each feature in the performance of their random forest classifier. In order to obtain Table 5, first we calculate and sum all the incremental prediction errors over all datasets. We then sort the resulting values in a non-increasing order and consider the top 10 features in the sorted list. We divide each value by the summation of the top 10 values and obtain a percentage value, which we refer to as “importance”, as shown in Table 5. The “effect” column in the table indicates the direction of proportionality of the features with the ICL usage. A positive (negative) effect means that the higher (lower) the feature value, the more likely ICL usage.

**Table 5:** Feature importances for prediction of ICL product usage.

Feature Importance Rankings		
Feature Name	Importance	Effect
No. of Active Products	25.74%	Positive
Mean Credit Score	13.25%	Positive
cluster+d−l−r	9.86%	Negative
Income	9.84%	Positive
radial+d−l−r	9.18%	Negative
spatial_cluster_regularity	8.46%	Positive
Average Balance	7.59%	Positive
spatial_radial_regularity	5.86%	Positive
weekly_regularity	5.71%	Positive
week+d+l+r	4.51%	Positive

We observe from Table 5 that the top 3 features having the highest impact on classifier performance are a) number of active products, b) mean of the credit scores and c) cluster+d−l−r, with positive, negative and negative effects on ICL product usage, respectively. Note here that the representation cluster+d−l−r refers to a feature with cluster-based bins and the sum of diversity scores minus loyalty scores minus regularity scores (please refer to S1 Table for a full list of features considered along with their descriptions).

We need to keep in mind that all bank customers can apply for the ICL product, but the bank needs to approve such an application, after reviewing the customer’s financial status and past activity. In our dataset, if a customer uses an ICL in month 12, this means that the customer has already passed the approval phase and taken out the loan. Therefore, observing 3 of the features in Table 5, namely the mean of the credit scores, income and average balance is fairly logical because these are typically included among the evaluation criteria considered by the bank for loan applications. Furthermore, we see that all three features have a positive effect on ICL product usage, which is also expected.

What is probably less clear or harder to interpret is the remaining features based on customer mobility signatures (e.g. cluster+d−l−r or radial+d−l−r). For instance, the feature cluster+d−l−r with a negative effect suggests that the higher the cluster-based diversity and/or the lower the cluster-based loyalty and regularity, the less likely a customer to use the ICL product. Conversely, customers with more stable lives, going to similar places geographically and on a regular basis are more likely to use this product. We can illustrate this behavior with a real customer from our database who is a male mid-aged person living in Istanbul, and who has used the ICL product. He has made all of his transactions in 4 clusters only (out of 50 across Turkey), and the largest share (68%) of his transactions occurs around his workplace while the second largest share (16%) around his home location. Furthermore, this person has a cluster diversity score of 0.452, a cluster loyalty score of 0.798, and a cluster regularity score of 0.863. In other words, this individual, with relatively low diversity, high loyalty and high regularity scores, has a reasonably predictable and recurring mobility behavior around his residence and workplace, which supports the finding we have regarding cluster+d−l−r. A similar effect is also observable with the remaining mobility-based features (e.g. radial+d−l−r and week+d+l+r) with varying degrees of importance.

Next, when we look at the cluster seeds resulting from cluster-based calculations of bins from a geographical point of view, we observe that most of the cluster seeds are spread almost equally across the country except the seeds in Istanbul. In fact, 8 of the 50 seeds are located in Istanbul. This is not a surprising result as 19% of Turkey’s population lives in Istanbul and most of the economic activity takes place in Istanbul relative to 80 other provinces [22].

Finally, the results of the final testing stage for the 3,000 untested customer subset are presented in Table 6. The results given in Table 6 show findings that are consistent with those presented above. Here we observe that our models are not overfitting and there is not a significant difference between the performances. We find this as a strong evidence that the features that have been selected and used by the best combination of FSE algorithms and classifiers act as strong predictors of the ICL product usage.

**Table 6:** Final testing performance measures for different datasets.

Performance Measures					
Dataset	AUC	Accuracy	Precision	Recall	F1
**D**	0.681	62.421	0.527	0.632	0.575
**11DM**	0.835	79.077	0.721	0.724	0.723
**11DF**	0.940	86.040	0.834	0.802	0.817
**11DFM**	0.942	87.199	0.860	0.786	0.821
**9DM**	0.821	80.246	0.787	0.618	0.693
**9DF**	0.928	82.872	0.858	0.777	0.815
**9DFM**	0.938	86.906	0.869	0.814	0.840
**6DM**	0.853	79.747	0.749	0.699	0.723
**6DF**	0.914	85.467	0.827	0.805	0.816
**6DFM**	0.955	85.635	0.853	0.844	0.848
**3DM**	0.893	79.488	0.751	0.705	0.727
**3DF**	0.916	86.072	0.845	0.802	0.823
**3DFM**	0.955	85.571	0.881	0.845	0.863

Below is an overall summary of our most important findings in this study:

- The performance ranking of feature sets is *DFM* ≥ *DF* ≥ *DM* ≥ *D*, regardless of the dataset type.
- The more recent and up-to-date the mobility features, the better the classifier performance; hence, 3*DFM* ≥ 6*DFM* ≥ 9*DFM* ≥ 11*DFM* and similarly, 3*DF* ≥ 6*DF* ≥ 9*DF* ≥ 11*DF* and 3*DM* ≥ 6*DM* ≥ 9*DM* ≥ 11*DM*.
- The effect of mobility features is more pronounced as more recent mobility data is used. That is, (3*DFM* − 6*FDM*) ≥ (6*DFM* − 9*FDM*) ≥ (9*DFM* − 11*FDM*).
- All customers are able to apply for ICL, but customers with higher mean credit score, income and average account balance are more likely get approved and use ICL.
- Customers with more steady life cycles are more likely to get approved by the bank for ICL and use the product.

## Conclusions

In this study, we analyze one-year transactional microdata from a commercial bank to explore how individual mobility signatures and financial indicators relate to the use of a target product of the bank. The results we obtain in our study highlight the importance of spatio-temporal mobility features as well as features for financial activity in predicting product usage. We consider the Individual Consumer Loan (ICL) product of the bank as the target product and determine the best performing feature selection/extraction and classification algorithms for 13 scenarios of data time frame and feature sets, to predict the usage of this product. Our results reveal several interesting observations. First, due to the nature of the ICL product application and approval process, features related to an individual’s financial status with the bank (e.g., average balance, mean credit score) are more pronounced. Second, we discover that customers with more steady lives (as reflected by their mobility signatures) are more likely to use the ICL product. We further discover that as the mobility information used in the analysis becomes more recent and up-to-date, the accuracy of the classification methods and the predictability of product usage get better. This implies the existence of a strong relationship between individual mobility behavior and ICL product usage.

To summarize, we find that mobility behavior is almost as influential as financial features in explaining the usage pattern for a bank’s ICL product. While the extension of our analysis and this finding to other products of a bank is subject to validation, this is clearly a finding to exploit for making product recommendation systems perform better. As future work, we plan to investigate the validity of this notion for other products and also implement these concepts in the product recommendation system of the bank we work with. Such an implementation will collect customer responses for banking product offerings, which could potentially provide a better validation and a better real-world demonstration of our findings.

## Supporting information

S1 Table Feature explanations. (PDF)
