# Supplementary material for: Customer mobility signatures and financial indicators as predictors in product recommendation
Source: PLoS One. 2018 Jul 27;13(7):e0201197. doi: 10.1371/journal.pone.0201197 (PMC6063431; doi:10.1371/journal.pone.0201197)
Supplement: S1 Table — (PDF) [file pone.0201197.s001.pdf]

Supporting information

S1 Table. Feature explanations.

Feature Number	Feature Name	Data Type	Feature Type
**X1**	Customer ID	Integer	Demographic
**X2**	Age	Double Precision	Demographic
**X3**	Education Status	Text	Demographic
**X4**	Gender	Text	Demographic
**X5**	Marital Status	Text	Demographic
**X6**	Job	Text	Demographic
**X7**	Income	Double Precision	Financial
**X8**	Bank Age	Double Precision	Financial
**X9**	Average of Risk Scores	Integer	Financial
**X10**	Variance In Risk Scores	Double Precision	Financial
**X11**	Last Risk Score	Integer	Financial
**X12**	Number of Active Products	Integer	Financial
**X13**	Total Number of Call Center Calls	Integer	Financial
**X14**	Number of Call Center Calls Last Month	Integer	Financial
**X15**	Number of Total Credit Cards	Integer	Financial
**X16**	Number of Other Bank's Products	Integer	Financial
**X17**	Bank Credit Card Limit	Integer	Financial
**X18**	Total Credit Card Limit	Integer	Financial
**X19**	Average Interval Between Transactions	Double Precision	Financial
**X20**	Variance of Intervals Between Transactions	Double Precision	Financial
**X21**	Average Balance of the Account	Double Precision	Financial
**X22**	Last Known Balance of the Account	Double Precision	Financial
**X23**	Average Amount of Auto Payments	Double Precision	Financial
**X24**	Number of Auto Payments	Integer	Financial
**X25**	Days Remaining to Last Payment Date	Integer	Financial
**X26-X34**	Average Expenses	Double Precision	Financial
**X35**	Variance of Expenses	Double Precision	Financial
**X36**	Monthly Total Expenses	Double Precision	Financial
**X37**	Mobile Banking Usage	Integer	Financial
**X38**	Total Number of Credit Card Transactions	Integer	Financial

Feature Number	Feature Name	Data Type	Feature Type
**X39**	Total Number of ATM Transactions	Integer	Financial
**X40**	hourly_diversity	Double Precision	Mobility
**X41**	hourly_loyalty	Double Precision	Mobility
**X42**	hourly_regularity	Double Precision	Mobility
**X43**	weekly_diversity	Double Precision	Mobility
**X44**	weekly_loyalty	Double Precision	Mobility
**X45**	weekly_regularity	Double Precision	Mobility
**X46**	spatial_radial_diversity	Double Precision	Mobility
**X47**	spatial_radial_loyalty	Double Precision	Mobility
**X48**	spatial_radial_regularity	Double Precision	Mobility
**X49**	spatial_cluster_diversity	Double Precision	Mobility
**X50**	spatial_cluster_loyalty	Double Precision	Mobility
**X51**	spatial_cluster_regularity	Double Precision	Mobility
**X52**	Home-Work Distance	Double Precision	Mobility
**X53**	Max Average Monthly Distance	Double Precision	Mobility
**X54**	Max Variance Monthly Distance	Double Precision	Mobility
**X55**	hour+d+l+r	Double Precision	Mobility
**X56**	hour+d+l-r	Double Precision	Mobility
**X57**	hour+d-l-r	Double Precision	Mobility
**X58**	hour+d-l+r	Double Precision	Mobility
**X59**	week+d+l+r	Double Precision	Mobility
**X60**	week+d+l-r	Double Precision	Mobility
**X61**	week+d-l-r	Double Precision	Mobility
**X62**	week+d-l+r	Double Precision	Mobility
**X63**	radial+d+l+r	Double Precision	Mobility
**X64**	radial+d+l-r	Double Precision	Mobility
**X65**	radial+d-l-r	Double Precision	Mobility
**X66**	radial+d-l+r	Double Precision	Mobility
**X67**	cluster+d+l+r	Double Precision	Mobility
**X68**	cluster+d+l-r	Double Precision	Mobility
**X69**	cluster+d-l-r	Double Precision	Mobility
**X70**	cluster+d-l+r	Double Precision	Mobility
**Y**	Target Product Activity	Integer	Financial

The table, which is constructed for the 9 month data period (9DFM) including months 3 to 11, contains all the features we extract from the bank dataset. The first

feature, X1, is the ID of the customer, which is not used as a feature during the prediction process. X2 is the age of the customer. X3 is the education status of the customer (e.g., high school, university). X4 is the gender of the customer. X5 is the marital status of the customer (e.g., single, married). X6 is related to the job of the customer (e.g., private, public). All the features so far contain demographic information about the customers and from now on till X39, the features contain financial information. X7 is the monthly income of the customer. X8 is the duration that the customer uses the bank in terms of years. X9 is the variance of the risk scores of the customer through the data period. X10 is the mean of the risk scores of the customer through the data period. X11 is the last risk score of the customer in the data period, which corresponds to the 11th month in our data. X12 is the number of products that the user actively uses. In the dataset, there are two different concepts about product usage; product activity and product ownership. The activity definition varies according to the product. For example, for retirement savings product, the activity status is directly equal to the ownership status. This is because when the customer stops using this product, automatically she also loses the ownership status. On the other hand, for some products like time deposits, an inactivity of consecutive 3 months turns active status to inactive. In our dataset, when we examine the activity and ownership columns in detail, we observe that, they mostly represent the same values with a minor variation (in 98% of the cases the activity and ownership values are the same; for the rest, the activity values is 1 or 2 less than the ownership values). Therefore, we eliminate the ownership column and continue with activity values.

X13 is the total number of call center calls the customer made. X14 is the total number of call center calls the customer made in the last month of the period, which corresponds to the 11th month. X15 is the customer's total number of credit cards including other banks. X16 is the number of products that the customer uses from other banks. X17 is the credit card limit of the customer in our bank. X18 is the total credit card limit of the customer including other banks' credit card limits. X19 is the average time between two consecutive transactions of the customer. X20 is the variance of these consecutive transaction intervals. X21 is the average cash in the customer's account during the time period. X22 is the amount of cash in the 11th month. X23 is the average amount that the customer pays via auto payments. X24 is the total number of auto payments that the customer uses. X25 is the due date of the last payment of credit card date. X26-X34 are the average monthly expenses during the periods. There are 9 monthly expenses in this data, because the table is constructed for a 9 month dataset. For instance, X26 and X31 indicate the average monthly expenses in 1st and 6th months, respectively. X35 is the variance of these monthly expenses. X36 is the sum of these monthly expenses. X37 indicates the total number of mobile banking usage of the customer. X38 is the total number of credit card transactions the customer performed during the period. X39 is the total number of ATM transactions the customer performed during the period.

We have mentioned all demographic and financial features so far and the remaining features are mobility features except the class label. X40, X41 and X42 represent customer's diversity, loyalty and regularity measures calculated according to the hourly bins of transactions. X43, X44 and X45 represent customer's diversity, loyalty and regularity measures calculated according to the weekly bins of transactions. X46, X47 and X48 represent customer's diversity, loyalty and regularity measures calculated according to the spatial-radial bins of transactions. X49, X50 and X51 represent customer's diversity, loyalty and regularity measures calculated according to the spatial-cluster bins of transitions. X52 is the Euclidean distance between the customer's home and work locations. X53 is the average of monthly maximum distances that the user has taken during each month. X54 is the variance of these monthly maximum

distances. X55-X70 values are the addition-subtraction combinations of all the diversity, loyalty and regularity values (X40-X51). For instance, week+d+l-r represents the summation of positive weekly diversity, positive weekly loyalty and negative weekly regularity. Y is the output variable (class label) of our study. Y indicates the active usage of the target product (ICL) in the 12th month for each customer. All the elements in the feature set are used to predict the class label except for the ID column.
